# Dopaminergic genetic variation and response to social comparative feedback when learning a motor sequence task

**DOI:** 10.1371/journal.pone.0354275

**Published:** 2026-07-29

**Authors:** Allison F. Lewis, Rachel Bohnenkamp, Jill Campbell Stewart

**Affiliations:** University of South Carolina, Department of Exercise Science, Columbia, South Carolina, United States of America; Shiga Medical Center, JAPAN

## Abstract

Positive social comparative feedback during motor skill practice is hypothesized to enhance motor learning by triggering a dopaminergic response. Individual differences in dopamine-related genes impact dopamine neurotransmission and may influence responsiveness to motor practice conditions that target dopaminergic pathways. The purpose of this study was to examine the impact of dopamine genotype on learning of a motor sequence task under two different feedback conditions: response time only feedback or response time with positive social comparison. Fifty-two adults practiced a joystick-based motor sequence task over two consecutive days. On Day 1, participants were randomized to receive either 1) response time only feedback (i.e., “You completed the block in 80 seconds”) or 2) positive social comparative feedback (i.e., “You completed the block in 80 seconds. You were faster than others”). Motor learning was assessed by retention performance, or the change in response time from the first block of Day 1 to the first block on Day 2. Saliva samples were used to genotype for dopamine receptors DRD1, DRD2 and DRD3 and COMT. Individual genes were scored (0–2) and summed to create a polygene score (0–8). Participants were then categorized as having Low (1–4) or High (5–8) dopamine neurotransmission. A significant interaction was found between time, summary polygene group and feedback group (p = 0.048). The Low dopamine group showed greater improvements with response time only feedback versus positive social comparative feedback. The High dopamine group showed similar improvements in response time regardless of feedback type. This suggests that feedback targeting dopaminergic pathways may not be beneficial for individuals with lower dopamine neurotransmission. Our findings suggest that dopaminergic genetics may impact the efficacy of positive social comparative feedback on motor learning in low dopamine genotypes.

## 1. Introduction

Dopamine is a key regulator of physiological processes involved in motivation, motor learning, and reward responsiveness [1–3]. Dopamine neurotransmission is highly complex, encompassing interacting phasic, tonic, excitatory, and inhibitory processes that are dynamically shaped by biological factors as well as individual experiences, environmental influences, and behavioral habits. Likewise, motivation, motor learning, and reward processing circuits are complex, involving multiple non-dopaminergic neural circuits and shaped by many factors. Individual biological differences in dopamine neurotransmission—partly determined by genetic variation in the form of single-nucleotide polymorphisms (SNPs)—is one factor of many than can influence processes related to motivation, motor learning and reward responsiveness [4,5]. SNPs, or substitutions of a nucleotide at a specific location in the genome, can affect the expression of dopamine receptors as well as the enzymes involved in synaptic dopamine degradation, ultimately leading to relatively higher or lower dopamine neurotransmission [6–13]. These biological effects can manifest as measurable differences in individual characteristics and behaviors. For example, polymorphisms associated with relatively lower dopamine neurotransmission are linked to poorer motor learning abilities [14] and lower sensitivity to reward [15–18]. However, most studies linking dopamine-related genetics to motor learning have been conducted under uniform task practice conditions, limiting understanding of whether dopamine-related genetic differences may differentially influence learning when motivational and reward-related features of practice are manipulated.

Many factors interact to shape dopamine neurotransmission including genetics and state-dependent influences, like arousal, anxiety, prior experiences, and physical activity. Genetic variation is one tractable contributor within the broader dopamine system. Polymorphisms in genes encoding for D1-type dopamine receptors (DRD1 and DRD5), D2-like dopamine receptors (DRD2, DRD3, and DRD4), dopamine transporter (DAT), and dopamine degradation enzyme (COMT) can influence dopamine transmission by affecting receptor expression, receptor efficacy, dopamine transport, and synaptic degradation of dopamine [7,8,10,13,19–22]. Given the complexity of the dopamine system, genetic influences on dopamine neurotransmission and related behaviors may not be well explained by only considering the contribution of a single gene. Polygene scores, that consider the additive effectives of multiple genes, may be better suited for understanding system-wide effects of genetics on a behavioral outcome, like motor learning [14,23,24]. Given that hypodopaminergic states from genetics are associated with deficits in motor learning combined with the known function of dopamine in motivation [3,14,16,25], various strategies have been tested to enhance dopamine levels to support motivation and motor skill learning.

Motor practice conditions can be altered to enhance motivational or rewarding properties or to target specific neural circuits. For example, nonpharmacological and nonmonetary approaches to increasing dopamine to promote motor learning, such as exercise or provision of rewarding feedback, are being explored for their potential to support motor learning through motivational and reward pathways [26–29]. One of these strategies is positive social comparative feedback, which is feedback that indicates to the learner that they are performing better than others. This type of feedback is hypothesized to enhance the learner’s expectancies, increase perceptions of positive outcome or reward, and trigger a dopaminergic response that enhances motor learning and boosts feelings of self-efficacy or perceived competence [27]. Given that social comparative feedback is hypothesized to target dopaminergic pathways in the brain, individual genetic differences in dopamine-related genes may impact the neural and behavioral response to such feedback.

Several studies have explored the impact of positive social comparative feedback on motor learning [28–32]. However, a recent meta-analysis showed no effect of positive social comparative feedback on motor learning with some studies identifying positive effects and others seeing no significant effects [33]. The variability in the effects reported to date could be, at least in part, due to the influence of individual genetic variation. Our previous neuroimaging work suggests that positive social comparative feedback engages reward pathways in the brain which function via dopaminergic neurotransmission [34]. As such, the central response to positive social comparative feedback (engagement of dopaminergic reward circuits) may differ based on genotype thereby affecting motor learning response to feedback targeting this pathway.

To date, the impact of dopamine-related genetic polymorphisms on the response to positive social feedback during motor practice has not been studied. Therefore, the purpose of this secondary data analysis was to examine the effect of dopamine-related genetic polymorphisms on the motor learning response to a feedback condition that is hypothesized to target the dopamine system (positive social comparative feedback). We hypothesized that genotypes associated with lower dopamine neurotransmission would experience less learning benefit from positive social comparative feedback. Because low dopamine genotypes are linked to reduced reward sensitivity, individuals with a relatively lower dopamine neurotransmission due to genotype were expected to show diminished retention performance gains when exposed to feedback designed to engage reward circuitry.

## 2. Materials and methods

### 2.1. Participants

The current study was a secondary analysis that combined data from two separate primary studies which included a total of 78 participants. Of the 78, 16 participants were part of a control group that did not receive any performance feedback. These participants were not included in the current analysis. Of the remaining participants, one participant was unable to produce the required volume of saliva for genotyping and nine participants were not able to be full genotyped for the four SNPs of interest. This left 52 right-hand dominant, nondisabled participants to be included in this analysis. Data for 28 participants was collected in an initial study (Study 1) [35], and data for 24 additional participants were collected in a subsequent study (Study 2) [34]. Data collection for these studies was completed between February 2020 and November 2021. A sensitivity analysis was performed in G*Power (version 3.1) for a mixed model analysis of variance (ANOVA) with interactions that included 8 groups (Study X Feedback X Genotype) and two repeated measures. The analysis indicated that a total sample size of 52 provided 80% power (α = 0.05) to identify moderate effects (f = 0.28).

To be included in Study 1 or Study 2, participants had to be age 18–40 years, right-hand dominant [36], and able to move their right arm and hand without pain or limitation. Individuals were excluded from both studies if they were taking medications that might impact dopamine neurotransmission or if they had a current medical diagnosis affecting dopamine neurotransmission. Since Study 2 included magnetic resonance imaging (MRI), participants were excluded from that study if MRI was contraindicated (e.g., metal implants). To describe baseline characteristics of the study sample, participants completed the Rosenberg Self-Esteem Scale [37] and the State Trait Anxiety Index [38] at baseline. Both studies followed the same screening process, randomization procedures, and survey outcomes procedures as well as employed the same motor task and feedback delivery/content. Saliva sample collection and related procedures were also standardized across studies. The primary distinction between protocols was the implementation of MRI in Study 2, which occurred immediately before and immediately after the Day 1 motor practice. As such, Day 1 motor practice occurred in a quiet room in the MRI center in Study 2 and in a quiet laboratory in Study 1. Both studies were conducted in accordance with the Declaration of Helsinki and approved by the University of South Carolina Institutional Review Board. All participants provided written informed consent before beginning the research study.

### 2.2. Experimental design and serial target task

The experimental design with detailed methods has been fully described previously [34,35]. Briefly, participants were randomized into one of three feedback groups: no performance feedback group (CONTROL), response time only feedback group (RT ONLY), and response time with positive social comparison group (RT + POS). Participants practiced a serial target task on two consecutive days, where the first day was motor skill practice/acquisition with group specific feedback and the second day was retention testing of motor skill learning (no feedback provided). At the end of the second session on Day 2, participants provided a saliva sample to allow for genotype assessment. The primary distinction between Study 1 and Study 2 is that Study 2 included only two feedback groups (RT ONLY and RT + POS) and acquired brain imaging during the first session. Only the RT ONLY and RT + POS groups were included in the current analysis as these two groups both received performance feedback during practice on Day 1.

**Fig 1:**
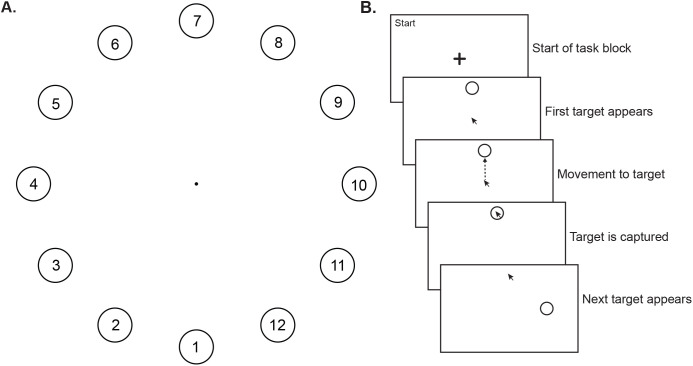
Schematic of joystick-based serial target task. **A)** Layout of all possible target locations, where the repeated sequence was 11-10-5-9-7-3-6-12. **B)** At the start of a block, a cross hatch appeared to indicate that the first target was about to appear. The mouse pointer indicated the position of the joystick and moved proportionally to the joystick movement. When the first target appeared, the participant moved the cursor inside of the target for 500 ms for the target to be “hit”. Once hit, the target disappeared and the next target appeared. The targets appeared one at a time in 8-target sequences without any pauses or cues between sequences. One block contained 5 sequences (40 targets). The sequences alternated between the repeated and random sequence type.

The serial target task (STT) was a joystick-based motor sequence task run in E-prime 2.0 (Psychology Software Tools, Inc., Sharpsburg, PA) (Fig 1A-B). Participants were seated at a desk with their dominant, right hand on the joystick. Displacement of the joystick was proportional to the movement of the cursor on a laptop screen. Participants manipulated the joystick with their right hand to guide a cursor inside 20 mm circular targets on the laptop screen (Fig 1A). The targets appeared one at a time in one of twelve distinct locations. To capture the target, the participant had to keep the cursor inside the target for 500 ms (Fig 1B). Participants were instructed to “hit” the series of circular targets as fast as they could.

The first session was organized into an exposure/baseline block followed by 28 blocks of practice. The exposure block contained five random order 8-target sequences intended to introduce the task and measure baseline motor performance. Each of 28 practice blocks consisted of five sequences that alternated between a random 8-target sequences and a stable, repeated 8-target sequence such that half of all practice sequences were random and half were a stable, repeated sequence. Performance on the repeated sequence was used for the investigation of implicit motor sequence learning; the random sequences were intended to keep the learner from explicitly identifying the repeated sequence. The random sequences were matched in difficulty to the repeated sequences by matching the total distance between all 8 targets in the sequence. After the first session, participants returned approximately 24 hours later for retention testing. Retention testing was identical to the first session, except no feedback was provided and only 12 blocks of the task were completed. The primary measure of performance and learning was response time or the total time to complete all 8 targets in the repeated sequence

During the first session of practice only, participants received their group specific feedback after every block. Feedback was provided in text on the laptop screen. The RT ONLY group received feedback about their response time to complete the block (e.g., “You completed this block in 91.3 seconds”). The RT + POS group received feedback about their response time plus positive social comparison indicating they were faster than others (e.g., “You completed this block in 91.3 seconds. You were 18.3 seconds faster than the average”). The social comparative difference (e.g., “You were 18.3 seconds faster than the average”) was calculated as a percentage of the individual participant’s response time on that block (14–20%, four blocks at 5%); therefore, this feedback was nonveridical but consistent in relative magnitude across participants [32].

Explicit awareness of the repeated sequence was tested in both studies at the end of Day 2 retention testing [34,35]. Explicit awareness of the repeated sequence was not common across studies with only 4/60 (6.66%) of participants able to reproduce part or all of the repeated sequence.

### 2.3. Genetic polymorphisms

Genetic information was collected from participants via saliva samples. Oragene collection kits (DNA Genotek, Ottawa, Ontario, Canada) were used to collect and store the saliva samples according to the manufacturer’s instructions. Participants were not permitted to eat or drink the hour prior to providing the sample, and this was strictly monitored during the session. All samples were immediately inspected by study personnel to ensure the required saliva sample volume was met. According to manufacturer recommendations, samples were tightly sealed and stored at ambient room temperature in a temperature-controlled building. Samples were also wrapped in black to felt to obscure light from reaching the samples. Under these conditions, samples remain stable for up to 5 years; none of our samples were stored for more than 2 years. Samples were shipped to and processed by Akesogen (Illumina Infinium HTS Assay with the Global Screening Array-24 v3.0; Norcross, GA; now Tempus, Chicago, IL) after the conclusion of all data collection. Participants with missing genotype calls at any of the four candidate SNPs were excluded from the final analysis (n = 9). The final analytic sample (n = 52) demonstrated high genotype completeness (mean call rate = 99.4%, range = 98.5–99.9%) and complete concordance between assay-determined and self-reported sex. Genotyping demonstrated strong overall call confidence, including favorable lower-tail confidence metrics (median p10 GenCall = 0.478) and central tendency (median p50 GenCall = 0.814).

From the genotyping results, we examined four dopamine-related single nucleotide polymorphisms (SNPs): three DA receptors (DRD1 rs4532, DRD2 rs1800497, DRD3 rs6280) and one DA degradation enzyme (COMT rs4680) (Table 1) as these were the set of dopamine-related SNPs available through standard assays. All genetic analyses were conducted by a third-party laboratory blinded to behavioral outcomes and study aims. Because genotype results were unavailable during behavioral testing, experimenters collecting motor outcomes were blinded to participants’ genetic status. Genetic and behavioral datasets were merged only at the final stage of analysis.

**Table 1:** Dopamine single nucleotide polymorphisms (SNPs).

SNP	Alleles	Description
**DRD1** *rs4532*	T/C	Known as A-48G with A to G substitution at position −48 and therefore, also a T to C substitution (G and C are complementary); presence of C allele is associated with increased greater D1 receptor binding [6,7,39,40]
***DRD2*** *rs1800497*	T/C	Known as ANKK1 TaqIA or Glu^713^Lys; C to T substitution; Presence of T allele associated with reduction in expression of D2 receptors and lower D2 binding [8,9,20,41–43]
**DRD3** *rs6280*	T/C	Known as Ser^9^Gly where the C allele encodes glycine and T allele encodes serine; C allele presence associated with greater D3 receptor binding [10,11,21,44,45]
**COMT** *rs4680*	A/G	Known as Val^158^Met polymorphism; G to A substitution results in methionine at position 158; A allele presence leads to lower enzyme degradation activity and increases dopamine synaptic availability [12,13,22,46,47]

T = thymine; C = cytosine; G = guanine; A = adenine.

Our primary analysis used a summary polygene score that included all four SNPs. Each SNP was scored based on both alleles from 0 (alleles corresponded to relatively lower dopamine expression) to 2 (alleles corresponded to relatively higher dopamine expression) (Table 1 and 2). A single, sum polygene score was calculated for each participant by summing the individual SNP scores. The result was a summary score that ranged from 0 to 8 (0 = low dopamine; 8 = high dopamine) [14,24]. Individuals were combined into a Low (summary score 1–4) and a High dopamine group (summary score 5–8) based on the polygene score. Collapsing across scores into dichotomized groups was required for model stability due to uneven distribution of participants across Genotype X Feedback groups (e.g., zero or low participant counts at extreme score polygene scores of 0, 1, 7, and 8) (See Table S1 in S1 File for distribution). Secondary exploratory analysis examined the effects of individual SNPs. For the individual SNP grouping, each participant was categorized into a Low or High group based on the known biological influence of the specific allele combinations (Table S2 in S1 File).

**Table 2:** Summary polygene scoring and grouping according to allele pair.

	*DRD1*	*DRD2*	*DRD3*	*COMT*
	*rs4532*	*rs1800497*	*rs6280*	*rs4680*
**0**	TT	TT	TT	GG
**1**	TC	TC	TC	AG
**2**	CC	CC	CC	AA

DRD1 = dopamine receptor D1; DRD2 = dopamine receptor D2; DRD3 = dopamine receptor D4; COMT = Catechol-O-methyltransferase; T = thymine; C = cytosine; G = guanine; A = adenine.

### 2.4. Statistical analysis

All statistical analyses were conducted in IBM SPSS Statistics version 28. Normality was assessed by Shapiro-Wilk test and visual inspection of histograms. A one-way ANOVA was run on non-categorical baseline data to determine any between group differences in demographics, psychosocial assessments, and motor task performance.

The primary analysis focused on the total polygene group in accordance with Table 2. Individual SNP groupings were explored in separate models (see Supplemental Methods in S1 File for details). To determine the effect of dopamine-related genetic variation on the motor learning response to feedback, we performed a repeated measures ANOVA to examine the effects of time (Day 1 Block 1, Day 2 Block 1), feedback group (RT ONLY, RT + POS), polygene group (Low, High), and their interactions on response time. We also included Study (1 or 2) as a factor to control for any study-related effects. To determine the locus of effects, we performed post-hoc analyses by conducting repeated measures ANOVAs separately within each genotype group to examine the effects of time, feedback group, and their interaction. For all analyses, significance was set at p < 0.05 and partial eta squared (η_p_^2^) was used to estimate effect sizes. For both the primary and post-hoc analyses, effects sizes were interpreted according to the following criteria: 0.01–0.059 = small, 0.06–0.139 = medium, and ≥ 0.14 = large [48].

## 3. Results

### 3.1. Participants

Participants were mostly female (38F/14M) and on average 26.1 ± 5.3 years old. The four polygene by feedback type groups (RT ONLY Low Dopamine, RT + POS Low Dopamine, RT ONLY High Dopamine, RT + POS High Dopamine) did not differ in baseline characteristics like age, self-esteem, state anxiety, trait anxiety, and baseline motor performance (Table 3). Similarly, the four groups for each individual SNP grouping did not differ in baseline characteristics (Tables S3-S6 in S1 File).

**Table 3:** Participant demographics and baseline characteristics for summary score groupings.

	LOW Dopamine		HIGH Dopamine	
	RT ONLY	RT + POS	RT ONLY	RT + POS
***n***	15	13	9	15
***Sex***	12F	10F	5F	11F
***Age (y)***	28.0 (6.1)	25.5 (4.9)	26.2 (6.1)	24.6 (3.9)
***Ethnicity***	14W/1A	9W/2A/1BR/1H	6W/2A/1BR	14W/1AA
***State Anxiety***	29.5 (10.2)	29.5 (6.7)	25.1 (5.1)	25.7 (3.9)
***Trait Anxiety***	33.5 (8.4)	32.5 (5.7)	30.0 (4.8)	29.9 (9.5)
***Rosenberg*** ***Self-Esteem***	34.6 (3.9)	35.5 (3.3)	35.9 (2.7)	35.7 (4.7)
***Baseline RT (s)***	15.3 (2.1)	14.0 (1.3)	14.7 (0.8)	15.0 (2.2)

Mean value (standard deviation); W = White/Caucasian (U.S., Canada, Europe, North Asia, Non-Hispanic); A = Asian; BR = Bi-racial or other; H = Hispanic/Latino (North American, Central American, South American); AA = African-American/Black (Non-Hispanic); RT = Response Time to complete 8-target sequence; No significant difference between groups on any baseline variable.

### 3.2. Genetic polymorphisms and motor learning

**Fig 2:**
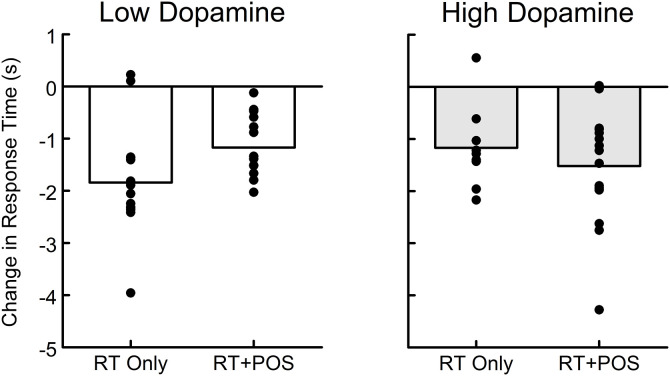
Change in response time from Day1, Block 1 to Day 2, Block 1 by feedback and summary gene score group to demonstrate the interaction between feedback group, polygene summary group, and time. Bars represent the group mean; circles represent individual participants. A negative change in response time represents faster performance of the repeated sequence at retention.

A significant main effect of time was observed (F_(1,44)_ = 96.814, p < 0.001, η_p_^2^ = 0.688), indicating faster response times at retention testing (Day 2, Block 1) and confirming that motor sequence learning occurred (Table 4). The effect of time did not differ based on feedback condition or genotype group alone (Time X Feedback group F_(1,44)_ = 0.108, p = 0.744, η_p_^2^ = 0.002; Time X Polygene group F_(1,44)_ = 0.403; p = 0.529; η_p_^2^ = 0.009). However, a significant interaction between time, feedback group, and polygene group showed that dopamine summary genotype group (Low, High) impacted motor learning response to the type of feedback provided (Fig 2; Time X Feedback X Polygene group interaction F_(1,44)_ = 4.327, p = 0.043, η_p_^2^ = 0.090). Post hoc testing revealed an interaction between time and feedback group for the Low Dopamine group (F_(1,24)_ = 5.143, p = 0.033, η_p_^2^ = 0.176) but not for the High Dopamine Group (F_(1,20)_ = 0.944, p = 0.343, η_p_^2^ = 0.045). In the Low Dopamine Group, the RT ONLY group showed a greater reduction in response time from Day 1 Block 1 (mean = 13.102, SE = 0.248, 95% CI [12.591, 13.613]) to retention (mean = 11.302, SE = 0.190, 95% CI [10.910, 11.694]) than did the RT + POS group from Day 1 Block 1 (mean = 12.428, SE = 0.273, 95% CI [11.865, 12.991]) to retention (mean = 11.301, SE = 0.209, 95% CI [10.869, 11.733]) (Table 4).

**Table 4:** Estimated marginal means of response time (seconds) for the repeated sequence from post-hoc repeated measures GLM.

	First Block of Practice		Retention Test	
	*Mean (Std. Error)*	*95% CI*	*Mean (Std. Error)*	*95% CI*
***LOW Dopamine***				
*RT ONLY*	13.10 (0.25)	12.59-13.61	11.30 (0.19)	10.91-11.69
*RT + POS*	12.43 (0.27)	11.87-12.99	11.30 (0.21)	10.87-11.73
***HIGH Dopamine***				
*RT ONLY*	12.16 (0.52)	11.08-13.24	11.12 (0.36)	10.37-11.86
*RT + POS*	12.55 (0.34)	11.85-13.26	11.02 (0.24)	10.53-11.51

First block of practice = block 1 on day 1; Retention test = block 1 on day 2; RT ONLY = response time only feedback group; Std. Error = standard error; 95% CI = 95% confidence interval as lower bound-upper bound.

For the exploratory analysis of single SNP groupings, a significant main effect of time was observed (p < 0.001) demonstrating that response times were faster at retention testing. The three-way interaction between Time, Feedback Group, and Single Gene Score was not significant (p > 0.074) for any analysis. Full results can be found in the Supplement (Tables S7 to S11 in S1 File).

## 4. Discussion

The current study identified a significant interaction between time (Block 1 of practice on Day 1, Block 1 of retention testing on Day 2), dopamine summary gene score (Low, High) and feedback type (response time only, response time with positive social comparison) suggesting that genotype may moderate the motor sequence learning response to the performance feedback provided. The group with relatively lower dopamine neurotransmission based on genotype (Low Dopamine group) showed less improvement in response time when provided positive social comparative feedback (1.1 seconds on average) compared with response time only feedback (1.8 seconds on average). The group with relatively higher dopamine neurotransmission based on genotype (High Dopamine group) showed similar improvement in both feedback conditions. Positive social comparative feedback, which is hypothesized to engage dopaminergic pathways, may be less supportive of motor sequence learning in individuals with low dopamine neurotransmission due to genotype.

Positive social comparative feedback is one practice condition that has been proposed to support motor learning [27]. Expectancy enhancing practice conditions, such as positive social comparative feedback, are hypothesized to support the learner’s belief that they will be successful on their next practice trial [14,49]. These practice conditions are believed to support motor performance and motor learning by increasing learners’ motivation towards a task, since humans have a basic need to feel competent and are motivated to engage in behaviors that result in success or reward [27]. However, recent metanalyses have highlighted some controversies in the field and called into question the effectiveness of expectancy enhancing practice conditions [33,50]. Only about half of studies investigating social comparative feedback demonstrated significant positive effects on motor learning [33]. While there are several potential methodological considerations that could explain the mixed findings, our results suggest that genetic variation could, at least in part, explain differences in motor learning responses to social comparative feedback and may contribute to the null effects identified in some of these previous studies.

The results of the current study provide preliminary evidence that genotype may influence the motor learning response to a practice condition intended to target dopaminergic pathways. Low dopamine genotypes have been associated with detrimental effects on learning, including motor sequence learning [14,49]. As such, methods for enhancing dopamine neurotransmission during motor skill practice are being investigated. In previous studies, combining medication or exercise with motor practice counteracted the effects of lower dopamine genotypes and motor learning was improved [14,26]. Our findings diverge from this idea that dopamine boosting strategies are beneficial for those with low dopamine genotypes. In the current study, the low dopamine group did not benefit from social comparative feedback and instead showed poorer motor learning effects with the rewarding feedback type. However, there are potential differences in the central nervous system effects of rewarding feedback versus interventions like exercise or medications. For example, medication or exercise results in diffuse, tonic neurotransmitter release while positive social comparative feedback likely triggers a phasic dopamine release similar to receipt of a reward [51–53]. While this is speculative in nature, it is possible that diffuse, tonic release of dopamine via medication or exercise enhances the ability of a low-capacity system by making dopamine available to any available dopamine receptor over a longer timescale thereby minimizing the effect of fewer receptors or high-degradation activity. On the other hand, consistent with previous studies shows that low dopamine genotypes have lower sensitivity to reward [15–18], an intervention that relies on event-triggered phasic release of dopamine may be limited in its effect when the available dopamine receptors are fewer and dopamine release occurs in brief, phasic events that do not leave dopamine available in the synapse for sustained timeframes. However, these ideas are speculative and require additional studies to disentangle these complex relationships, since the current study did not directly assess neurochemical or physiological measures.

The summary gene score was utilized for the primary analysis similar to previous studies [14,24], and our sample size has power to detect moderate effects for the Time X Feedback Group X Genotype interaction in this primary analysis. Consistent with this, we observed a significant three-way interaction for the polygene grouping, but not for the exploratory individual genotype groupings. Effect size estimates should therefore be interpreted cautiously given the limited precision and the possibility that smaller effects were not detectable. Genotypes were collapsed and dichotomized into high and low groups due to the available sample size and the uneven distribution of participants across genotype X feedback groups. While this approach improved model stability, collapsing categories reduced information, may attenuate power, and limits biological interpretability. Also, the scoring approach assumes comparable directional and magnitude influence of each genotype and does not capture potential interactive effects.

There are some considerations when interpreting the results of the current study. First, our conclusions are based on the observed significant interaction rather than large absolute performance differences. Findings should be interpreted within the context that we do not have defined values for what constitutes meaningful differences in response times on this task. Second, our analysis generated genotype scores based on four dopamine related SNPs that code for dopamine receptors (DRD1, DRD2, DRD3) and the dopamine degradation enzyme COMT. However, we were not able to assess every possible genetic variation that could impact dopamine neurotransmission (such as those impacting DRD4 receptors, DRD5 receptors, or DAT) and focused on those that had been assessed in previous motor learning work. Additionally, while we excluded participants with medication use or diagnoses known to impact dopamine, we did not account for other possible influences on dopamine such as environmental factors, physical activity, caffeine use, nicotine use, sleep, stress, or subclinical mood symptoms which could have influenced our outcomes and limited mechanistic interpretability. Third, the generalizability of our findings is limited by our sample population (right-handed, nondisabled, and relatively young (18–40 years)), our focus on the dopamine system (and not other systems), and the duration of practice. The identified effects of genotype on response to feedback type may not be the same in an older sample, may not occur in non-dopaminergic pathways, and may vary if longer practice duration was provided. While genotype is stable, the expression of genotype is impacted by aging and may lead to significant genotype by age interactions [23,54]. Additionally, there is evidence that aging can magnify the negative consequences of a genotype [55] or weaken effects of genetics that are evident in younger samples [56]. Fourth, the task and feedback should be considered in interpretation. The social comparative feedback was nonveridical and based on the individual participant’s performance on the block to ensure the relative magnitude of the feedback was consistent across participants. However, we were not able to control for an individual’s perception of the feedback. Participants may or may not have perceived the feedback as credible. The current study employs a laboratory-based motor task, and interpretations may be limited by the measure of motor learning (response time on a laboratory-based motor sequence task). Finally, the current study was powered to identify moderate effects which limits precision of estimates of effect size as well as confidence intervals.

## 5. Conclusion

The results suggest that dopamine-related genetic polymorphisms may interact with feedback type to affect motor sequence learning response. The group with a genotype resulting in low dopamine neurotransmission showed less improvements in response time when provided positive social comparative feedback compared to performance feedback alone. The group with a genotype resulting in high dopamine neurotransmission performed equally well under both feedback conditions. Confirmation with larger samples designed to more fully characterize genotype-by-feedback interactions in motor learning are needed. Future work might also consider additional measures to better understand underlying mechanisms.

## Supporting information

S1 File Supplemental materials. (PDF)
