# Supplementary material for: Dopaminergic genetic variation and response to social comparative feedback when learning a motor sequence task
Source: PLoS One. 2026 Jul 29;21(7):e0354275. doi: 10.1371/journal.pone.0354275 (PMC13419189; doi:10.1371/journal.pone.0354275)
Supplement: S1 File — (PDF) [file pone.0354275.s001.pdf]

Supplemental Materials

Dopaminergic genetic variation and response to social comparative feedback when learning a motor sequence task

Allison F. Lewis[1], Rachel Bohnenkamp[1], and Jill Campbell Stewart[1*]

[1]University of South Carolina, Department of Exercise Science, Columbia, South Carolina, USA

*** Correspondence:**
Jill Campbell Stewart
jcstewar@mailbox.sc.edu

Methods

Participant Distribution across Continuous Polygene Scores

Table S1. Frequency distribution of participants with each summary gene score by feedback group

Summary Gene Score	Feedback Group	Count
**0**	RT Only	0
	RT+POS	0
**1**	RT Only	0
	RT+POS	1
**2**	RT Only	3
	RT+POS	3
**3**	RT Only	5
	RT+POS	5
**4**	RT Only	7
	RT+POS	4
**5**	RT Only	6
	RT+POS	13
**6**	RT Only	3
	RT+POS	1
**7**	RT Only	0
	RT+POS	1
**8**	RT Only	0
	RT+POS	0

Individual SNP Analysis

For the secondary exploratory analysis of individual SNP grouping, each participant was categorized into a Low or High group based on the known biological influence of the specific allele combinations (Table 1 and Table S2). Repeated measures general linear models (GLM) were utilized to examine the effects of time (Day 1 Block 1, Day 2 Block 1), feedback group

(RT ONLY, RT+POS), individual gene score (Low, High), and their interactions on response time. Significance was set at $p<0.05$ and partial eta squared (η_p^2) was used to estimate effect sizes.

Table S2: Single SNP Grouping according to Allele Pair

	DRD1 rs4532	***DRD2 rs1800497***	***DRD3 rs6280***	***COMT rs4680***
**LOW**	TT	TT/TC	TT	GG
**HIGH**	TC/CC	CC	TC/CC	AG/AA

DRD1 = dopamine receptor D1 ; DRD2 = dopamine receptor D2; DRD3 = dopamine receptor D4; COMT = Catechol-O-methyltransferase; T = thymine; C = cytosine; G = guanine; A = adenine

Participants

For secondary exploratory analysis, participants were grouped based on single gene score. Sample characteristics based on these groupings can be found in Tables S3-6. The groups for each individual SNP grouping did not differ in baseline characteristics.

Table S3. Participant Demographics and Baseline Characteristics for Individual Gene Score Grouping (DRD1)

	**LOW Dopamine**		**HIGH Dopamine**	
	**RT ONLY**	**RT+POS**	**RT ONLY**	**RT+POS**
n	8	9	16	19
Sex	6F	7F	11F	14F
Age (y)	27.5 (6.9)	25.9 (5.0)	27.2 (5.8)	24.6 (4.0)
Ethnicity	7W/1A	6W/1AA/1A/1H	13W/2A/1BR	17W/1A/1BR
State Anxiety	29.8 (13.2)	29.8 (7.9)	26.9 (5.9)	26.4 (4.0)
Trait Anxiety	31.8 (9.6)	35.1 (10.8)	32.4 (6.3)	29.2 (5.5)
Rosenberg Self-Esteem	34.1 (4.6)	34.9 (5.2)	35.6 (2.8)	36.0 (3.5)
Baseline RT (s)	15.6 (2.4)	15.0 (2.4)	14.8 (1.3)	14.3 (1.7)

Table S4. Participant Demographics and Baseline Characteristics for Individual Gene Score Grouping (DRD2)

	LOW Dopamine		HIGH Dopamine	
	RT ONLY	RT+POS	RT ONLY	RT+POS
n	9	10	15	18
Sex	5F	6F	12F	15F
Age (y)	25.3 (5.3)	26.3 (4.6)	28.6 (6.3)	24.3 (4.2)
Ethnicity	8W/1A	9W/1A	12W/2A/1BR	14W/1A/1AA/1BR/1H
State Anxiety	28.3 (6.4)	30.3 (7.1)	27.5 (10.1)	25.9 (4.0)
Trait Anxiety	31.7 (7.6)	31.8 (7.4)	32.5 (7.4)	30.7 (8.4)
Rosenberg Self-Esteem	34.3 (3.2)	35.8 (3.6)	35.5 (3.6)	35.5 (4.4)
Baseline RT (s)	15.3 (2.3)	13.4 (0.8)	14.9 (1.4)	15.1 (2.1)

Table S5. Participant Demographics and Baseline Characteristics for Individual Gene Score Grouping (DRD3)

	LOW Dopamine		HIGH Dopamine	
	RT ONLY	RT+POS	RT ONLY	RT+POS
n	13	8	11	20
Sex	10F	6F	7F	15F
Age (y)	25.8 (5.2)	27.0 (4.7)	29.1 (6.6)	24.2 (4.0)
Ethnicity	12W/1A	6W/1A/1BR	8W/2A/1BR	17W/1AA/1A/1H
State Anxiety	30.8 (10.4)	27.9 (7.9)	24.4 (4.8)	27.3 (4.6)
Trait Anxiety	34.1 (6.9)	31.6 (7.3)	29.9 (7.5)	30.9 (8.3)
Rosenberg Self-Esteem	33.7 (3.8)	35.1 (3.9(	36.7 (2.1)	35.8 (4.2)
Baseline RT (s)	15.3 (2.0)	14.3 (1.5)	14.7 (1.4)	14.6 (2.1)

Table S6. Participant Demographics and Baseline Characteristics for Individual Gene Score Grouping (COMT)

	LOW Dopamine		HIGH Dopamine	
	**RT ONLY**	**RT+POS**	**RT ONLY**	**RT+POS**
n	7	10	17	18
Sex	7F	8F	10F	13F
Age (y)	29.8 (6.9)	24.7 (4.7)	26.3 (5.5)	25.2 (4.2)
Ethnicity	7W	8W/1A/1H	13W/3A/1BR	15W/1AA/1A/1BR
State Anxiety	28.6 (5.7)	29.3 (5.8)	27.5 (9.9)	26.4 (5.4)
Trait Anxiety	34.9 (5.6)	30.9 (5.6)	31.1 (7.8)	31.2 (9.1)
Rosenberg Self-Esteem	36.0 (2.2)	35.0 (3.0)	34.7 (3.9)	35.9 (4.6)
Baseline RT (s)	14.8 (1.8)	14.6 (1.4)	15.1 (1.7)	14.4 (2.2)

Mean value (standard deviation); W = White/Caucasian (U.S., Canada, Europe, North Asia, Non-Hispanic); A = Asian; BR = Bi-racial or other; H = Hispanic/Latino (North American, Central American, South American); AA = African-American/Black (Non-hispanic); RT = Response Time to complete 8-target sequence; No significant difference between groups on any baseline variable

Results

We identified a significant effect of time in all single gene group models (p<0.001) showing that response times got faster, regardless of single dopamine group. Dopamine single genotype group (Low, High), feedback group (RT only, RT+POS), or their interaction did not have significant effects on response time for any of the individual SNPs (Table S7).

Table S7. Single gene group general linear model results – within-subjects effects while controlling for Study

	Source	***F***	***p-value***	η_p^2
DRD1	Time	130.106	<0.001	0.747
	Time x Feedback Group	0.046	0.831	0.001
	Time x Single Gene Group	0.117	0.734	0.003
	Time x Feedback Group x Single Gene Group	2.604	0.114	0.056
DRD2	Time	116.988	<0.001	0.995

	Time x Feedback Group	3.419	0.071	0.072
	Time x Single Gene Group	3.200	0.081	0.068
	Time x Feedback Group x Single Gene Group	3.243	0.079	0.069
DRD3	Time	99.489	<0.001	0.693
	Time x Feedback Group	0.729	0.398	0.016
	Time x Single Gene Group	0.650	0.425	0.015
	Time x Feedback Group x Single Gene Group	3.333	0.075	0.070
COMT	Time	135.840	<0.001	0.755
	Time x Feedback Group	4.191	0.047	0.087
	Time x Single Gene Group	4.073	0.050	0.085
	Time x Feedback Group x Single Gene Group	3.291	0.076	0.070

Table S8. Estimated marginal means of response time (seconds) for the repeated sequence – DRD1

	**First Block of Practice**		**Retention Test**	
	Mean (Std. Error)	*95% CI*	*Mean (Std. Error)*	*95% CI*
LOW Dopamine				
RT ONLY	12.70 (0.40)	11.90-13.50	11.41 (0.28)	10.85-11.97
RT+POS	13.09 (0.40)	12.29-13.89	11.43 (0.28)	10.86-11.99
HIGH Dopamine				
RT ONLY	13.00 (0.29)	12.41-13.58	11.18 (0.20)	10.77-11.59
RT+POS	12.32 (0.26)	11.80-12.85	11.00 (0.18)	10.63-11.37

First block of practice = block 1 on day 1; Retention test = block 1 on day 2; RT ONLY = response time only feedback group; Std. Error = standard error; 95% CI = 95% confidence interval as lower bound-upper bound

Table S9. Estimated marginal means of response time (seconds) for the repeated sequence – DRD2

	First Block of Practice		Retention Test	
	Mean (Std. Error)	*95% CI*	*Mean (Std. Error)*	*95% CI*
LOW Dopamine				
RT ONLY	13.18 (0.36)	12.44-13.91	11.53 (0.27)	10.98-12.07
RT+POS	11.85 (0.35)	11.14-12.55	11.15 (0.26)	10.62-11.67
HIGH Dopamine				
RT ONLY	12.75 (0.29)	12.18-13.33	11.11 (0.21)	10.68-11.53
RT+POS	12.82 (0.26)	12.30-13.34	11.18 (0.19)	10.80-11.57

First block of practice = block 1 on day 1; Retention test = block 1 on day 2; RT ONLY = response time only feedback group; Std. Error = standard error; 95% CI = 95% confidence interval as lower bound-upper bound

Table S10. Estimated marginal means of response time (seconds) for the repeated sequence – DRD3

	First Block of Practice		Retention Test	
	Mean (Std. Error)	*95% CI*	*Mean (Std. Error)*	*95% CI*
LOW Dopamine				
RT ONLY	13.03 (0.33)	12.38-13.69	11.15 (0.22)	10.70-11.60
RT+POS	12.14 (0.43)	11.45-13.18	11.56 (0.27)	11.01-12.11
HIGH Dopamine				
RT ONLY	12.71 (0.40)	11.91-13.51	11.56 (0.27)	11.01-12.11
RT+POS	12.58 (0.26)	12.05-13.11	11.16 (0.18)	10.80-11.52

First block of practice = block 1 on day 1; Retention test = block 1 on day 2; RT ONLY = response time only feedback group; Std. Error = standard error; 95% CI = 95% confidence interval as lower bound-upper bound

Table S11. Estimated marginal means of response time (seconds) for the repeated sequence – COMT

	**First Block of Practice**		**Retention Test**	
	Mean (Std. Error)	*95% CI*	*Mean (Std. Error)*	*95% CI*
LOW Dopamine				
RT ONLY	13.72 (0.46)	12.79-14.65	11.27 (0.330)	10.61-11.94
RT+POS	12.93 (0.35)	12.23-13.63	11.56 (0.25)	11.06-12.06
HIGH Dopamine				
RT ONLY	12.68 (0.27)	12.14-13.22	11.31 (0.19)	10.92-11.69
RT+POS	12.28 (0.26)	11.75-12.80	10.97 (0.19)	10.59-11.34

First block of practice = block 1 on day 1; Retention test = block 1 on day 2; RT ONLY = response time only feedback group; Std. Error = standard error; 95% CI = 95% confidence interval as lower bound-upper bound
